# AI-based betting anomaly detection system to ensure fairness in sports and prevent illegal gambling

**DOI:** 10.1038/s41598-024-57195-8

**Published:** 2024-03-18

**Authors:** Changgyun Kim, Jae-Hyeon Park, Ji-Yong Lee

**Affiliations:** 1 Department of Artificial Intelligence & Software, Kangwon National University, Samcheok, 25913 Republic of Korea; 2 Center for Sports and Performance Analysis, Korea National Sport University, Seoul, 05541 Republic of Korea

**Keywords:** Logistic regression, Random forest, Support vector machine, k-Nearest neighbor, Ensemble model, Match-fixing, Engineering, Mathematics and computing, Computational science, Computer science, Information technology, Software

## Abstract

This study develops a solution to sports match-fixing using various machine-learning models to detect match-fixing anomalies, based on betting odds. We use five models to distinguish between normal and abnormal matches: logistic regression (LR), random forest (RF), support vector machine (SVM), the k-nearest neighbor (KNN) classification, and the ensemble model—a model optimized from the previous four. The models classify normal and abnormal matches by learning their patterns using sports betting odds data. The database was developed based on the world football league match betting data of 12 betting companies, which offered a vast collection of data on players, teams, game schedules, and league rankings for football matches. We develop an abnormal match detection model based on the data analysis results of each model, using the match result dividend data. We then use data from real-time matches and apply the five models to construct a system capable of detecting match-fixing in real time. The RF, KNN, and ensemble models recorded a high accuracy, over 92%, whereas the LR and SVM models were approximately 80% accurate. In comparison, previous studies have used a single model to examine football match betting odds data, with an accuracy of 70–80%.

## Introduction

Sports events take place in an environment of fair competition among competitors that is governed by rules for each game and professional referees that make fair judgments^1,2^. In a fair competitive environment, game results are determined by internal factors related to the athletes, including physical ability, effort, and conditions, as well as external factors, such as chance, weather, field conditions, and referee standards^3^. The public watches sports enthusiastically because of the excitement and uncertainty of the results under various conditions and the belief that the players did their best under fair conditions. However, it is challenging for athletes to increase their competence and train to always perform at the highest level^4,5^. Efforts to ensure fairness in sports are ongoing. To ensure fairness and equal chances of winning for all contestants, regardless of different physical abilities, athletes are classified by gender and weight in some sports, and by age in others, to ensure equality of opportunity, regardless of differences in cognitive ability^6^.

Unfortunately, some people aim to predetermine sports results through illegal practices^7,8^. Typical illegal practices include “doping”—the use of banned substances, such as performance-enhancing drugs in competitive sports, and match-fixing—the act of playing or officiating a match with the intention of achieving a predetermined result by manipulating internal conditions, such as referees, opponents, or coaches^9,10^. There are various types of match-fixing, largely divided into those in the pursuit of financial gain and those involving human networks. The former involves athletes and brokers earning dividends by betting through a betting site, while the latter is conducted in pursuit of honor or advantage in entrance exams^11,12^. The most frequent type of match-fixing is related to financial gain. As an average professional athlete is likely to retire in their late thirties, they face uncertain economic futures and may feel tempted to take part in match-fixing as an easy way to make money^13^. Match-fixing in sports is emerging as a serious issue that damages the spirit of sports and has a substantially negative impact on the industry. Therefore, it is necessary to develop a system to detect match-fixing in sports.

Anomalies in sports refer to abnormal or unusual patterns or behaviors that deviate from the expected or typical. In the context of this study, anomalies would refer to suspicious activities or behaviors that indicate potential match-fixing. Match-fixing detection aims to identify and prevent activities that undermine the fairness and integrity of sports competitions. Anomalies play a crucial role in detecting match-fixing, as they can manifest in various forms, such as unusual betting patterns, unexpected performance fluctuations, or suspicious player behaviors. Anomalies serve as red flags that raise suspicions of potential match-fixing, and detecting them is essential. By analyzing these anomalies, we can uncover instances of manipulation and take appropriate action to maintain the fairness of sports competitions. This study aims to develop a system for detecting match-fixing in sports by leveraging an AI-based model and analyzing sports betting odds. By collecting and analyzing a comprehensive set of variables—sports results, team rankings, and player data—our system can identify anomalies that may indicate match-fixing activities. By integrating advanced technology and thorough data analysis, we aim to contribute to the eradication of match-fixing in sports and ensure integrity within sports.

## Literature review

### Market risks of match-fixing

Match-fixing in sports could create huge profits for those involved in corrupt activities; however, it has significant negative consequences, such as threatening the integrity of the sport and causing fans to leave. Although people love sports for various reasons, the excitement and uncertainty of the results are at the core of this love. As chance factors, such as player conditions during the game, influence the match result, the public is enthusiastic about sports and cheers for the athletes. If the match results are manipulated and predetermined, the public will abandon sports and athletes will lose their motivation to compete^14^.

Continued match-fixing could have a substantial negative influence on sports, and the industry will inevitably shrink. It is therefore crucial to detect anomalies and match-fixing to protect the future of sports and athletes.

### Detection of behaviors of athletes and those involved in match-fixing

Various studies have been conducted on match-fixing detection. Some focused on detection using the player behavior patterns. For instance, in 2014, a common-opponent stochastic model was developed to predict the outcome of professional tennis matches and identify match-fixing when anomalies arise in athletes’ behavior during games and betting^15^. Another study investigated the behavior of tennis players to detect match-fixing in games by examining the number of rallies between players to determine if they followed Banford’s law^16^. To assess the status of match-fixing to influence something other than betting, surveys were conducted to investigate factors such as school admission and coaches’ requests^17^.

### Match-fixing detection using betting odds and market price figures

The ability to detect match-fixing through the behavioral patterns of players that are influenced by contingent factors and players’ physical conditions is limited. Therefore, it is necessary to set an index that can predict game results to detect anomalies using sports game data. The index, which can predict game results and identify differences between competing teams, can be represented by the sports betting odds^18^. The betting odds are generated by considering a range of factors, including recent performance, game flow, match results, injured players, and penalized players. Strong teams receive low odds, whereas weak teams receive high odds. However, not all sports betting companies offer the same odds. How odds are determined is closely related to the margin set by the sports betting company; specifically, the odds vary depending on how much margin the betting company intends to retain. For instance, if the initial odds are set at 2.20 for a home team to win, 3.25 for a tie, and 3.30 for a loss, the company’s margin for a win would be 6%. Different odds are therefore generated, even for the same sports event, depending on the country or league of the betting company. The equations are as follows:

1 $$Home Team Win = \left( {\frac{1}{2.20} \times 100} \right) = 45\%$$

2 $$Home Team Draw = \left( {\frac{1}{3.25} \times 100} \right) = 31\%$$

3 $$Home Team Lose = \left( {\frac{1}{3.30} \times 100} \right) = 30\%$$

4 $$Total Margin = \left( {45\% + 31\% + 30\% } \right) = 106\%$$

Odds have often been used to determine the value of athletes and teams and to predict match results^19^. In a study on the detection of match-fixing, data were examined by monitoring various online betting sites in real time; match-fixing was determined when an irregular betting pattern occurred for the same game on a specific site^20^. Archontakis and Osborne^21^ detected match-fixing by analyzing the betting results of the 2002 World Cup soccer match using the Fibonacci sequence. Previous studies have also used data from the Sportradar Fraud Detection System, which detects match-fixing based on global betting activities for soccer games^22^. Other studies have attempted to detect match-fixing through the betting odds^23,24^. This method is considered effective for detecting match-fixing and is accepted by the Court of Arbitration for Sports as the main evidence in sports match-fixing cases^25,26^.

Continuous efforts have been made to build a system for detecting abnormal signals in sports. To eliminate cheating in sports, further efforts have promoted the introduction of monitoring systems^27^. In addition, as the odds pattern for match-fixing occurs at specific sites, the continuous data collection to identify match-fixing through these sites can be presented as a solution for eradicating sports match-fixing. This study proposes a solution to eliminate match-fixing in sports by building a database of a range of variables, including sports results, team rankings, and players, using an AI-based model to detect anomalies based on the sports betting odds.

## Materials and methods

This study aimed to build a sports betting database to ascertain anomalies and detect match-fixing through betting odds data. The database contains data on sports teams, match results, and betting odds. A match-fixing detection model was created based on the database.

### Sport database

The database was built on world football league match betting data of 12 betting companies (188bet, Interwetten, Vcbet, 12bet, Willhill, Macauslot, Sbobet, Wewbet, Mansion88, Easybet, Bet365, and Crown), using historical database documentation of iSports API. The latter provides a vast collection of data on players, teams, game schedules, and league rankings for every sports league, including football, basketball, baseball, hockey, and tennis. This study constructed a database using data on soccer matches. As shown in Table 1, 31 types of data were collected. iSports API is a sports data company that offers application programming interfaces (APIs) for accessing and integrating sports data into various platforms and applications. The API collects data from multiple sources using a combination of automated web scraping technology, data feeds, and partnerships with sports data providers. To extract data, web scraping techniques are utilized on sports websites, including official league and team sites, news platforms, and sports statistics portals. Once gathered, the data are aggregated and presented in a consistent and structured format. This involves standardizing data fields, normalizing data formats, and merging information from different sources to create comprehensive and unified datasets. Furthermore, quality assurance measures are employed by iSports API to ensure the accuracy and reliability of the collected data, enhancing its overall reliability. The data collected by iSports API comprise match betting data from various world football leagues, covering the period from 2000 to 2020, including data from leagues, such as the K-League, Premier League, and Primera Liga. The dataset contains odds for home matches, away matches, and ties, which are recorded at minute intervals throughout each match.

**Table 1:** Collected data.

S/N	Form	Description
1	Player	Player Profile
2	PlayerInTeam	Player Team Information
3	Team	Team Profile
4	Sclass	League & Cup Profile
5	SclassInfo	Country Team Profile
6	Schedule	Schedule & Results Data
7	DetailResult	Events during the Match (change, score, injury)
8	Company	Sports Betting Site Company
9	MultiLetGoal	Asian Handicap
10	MultiLetGoalDetail	Asian Handicap (changes over time)
11	MultiLetGoalhalf	Asian Handicap Half-Time
12	MultiLetGoalhalfDetail	Asian Handicap Half-Time (changes over time)
13	MultiTotalScore	Over/Under
14	MultiTotalScoreDetail	Over/Under (changes over time)
15	MultiTotalScorehalf	Half-Time Over/Under
16	MultiTotalScorehalfDetail	Half-Time Over/Under (changes over time)
17	Standard	Win-Tie-Loss
18	StandardDetail	Win-Tie-Loss (changes over time)
19	StandardHalf	Half-Time Win-Tie-Loss
20	StandardHalfDetail	Half-Time Win-Tie-Loss (changes over time)
21	EuropeCompany	Data 200 + European sports betting sites
22	EuropeOdds	Win-Tie-Loss of 200 + European sports betting sites
23	EuropeOddsDetail	Win-Tie-Loss of 200 + European sports betting sites (changes over time)
24	EuropeOddsTotal	Win-Tie-Loss of 200 + European sports betting sites (average)
25	Score	League Ranking
26	CupMatch_Grouping	Cup Ranking
27	CupMatch	Final Cup Ranking
28	SubSclass	Playoffs
29	TeamTechStatistics	Team Statistics
30	PlayerTechStatistics	Player Statistics
31	PlayerTranslate	Player Position & Staff Role

**Figure 1:**
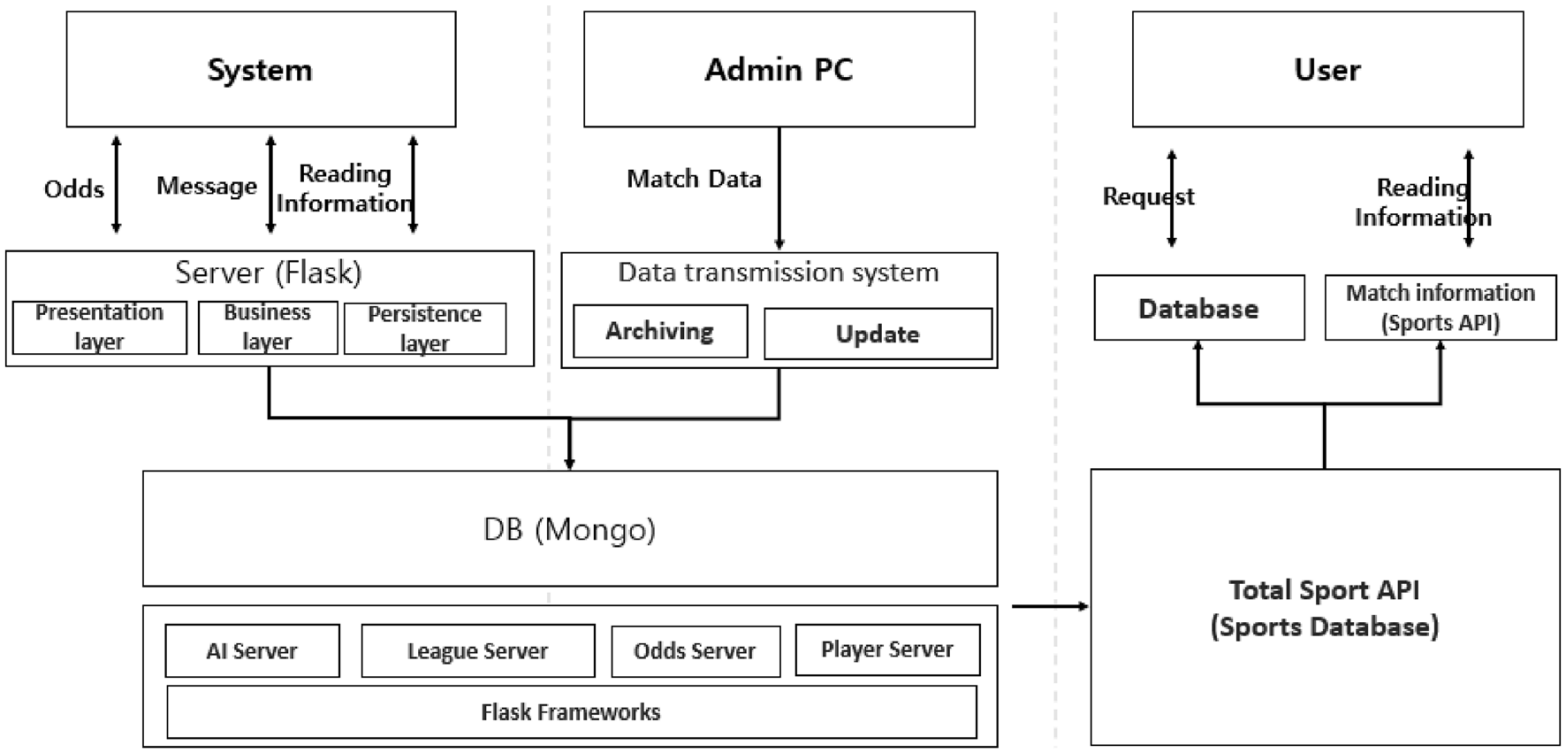
Database diagram.

The variables in Table 1 constitute the database, as shown in Fig. 1. The Flask server is available for users to request data on betting odds, user messages, and matches. The Admin PC constantly updates match data and stores them in the database. Database building took place in Mongo DB, providing the following servers: Sport Server on matches and weather; League Server on league and cup profiles, league ranking, and events during matches; Odds Server on betting odds of different categories as well as on betting company site; and Player Server on player’s performance, profile, and other information. The database, illustrated in Fig. 1, continuously collects soccer match data, based on 31 variables that have an impact on the outcome of the game. This allows us to assess whether the derived match odds exhibit a normal or abnormal pattern, based on various factors. The database also enables the comparison of real-time data on 31 variables and odds, thereby enabling the identification of abnormal games—both in real time and retrospectively.

### Betting models

This study employed four models: support vector machine (SVM), random forest (RF), logistic regression (LR), and k-nearest neighbor (KNN), known for their robust performance in classifying normal and abnormal games based on win odds, tie odds, and lose odds patterns. Instead of solely relying on the patterns of normal and abnormal games identified by these four distinct machine-learning models, we further integrated them into an ensemble model by aggregating their parameters. By pooling the predictions of all five models (the original four plus the ensemble) through a voting mechanism, we categorized games into three distinct patterns: “normal,” “warning,” and “abnormal,” based on the collective consensus of these models, The betting model of this study can be described by Algorithm 1 as follows: A total of five models were used to detect abnormal games, including four individual machine learning models and one ensemble model. The ensemble model was based on the parameters of the other four models. To determine the authenticity of a game, the results of all five models were aggregated. Furthermore, a game was categorized into one of the following three classifications: “normal,” “warning,” or “abnormal,” based on the number of models that identified the game as potentially fraudulent. This comprehensive dataset allowed us to identify patterns associated with abnormal matches, thereby enabling the classification model to learn and distinguish between normal and abnormal labels. Hence, the classification model was employed as a means to effectively analyze and comprehend the intricacies within the dataset. The data used for classification were employed to identify patterns of win odds, tie odds, and loss odds observed in soccer matches, using the proposed method. These patterns were then converted into specific values and utilized in the classification process. Thus, a specific pattern of odds in soccer matches served as a model variable.

**Figure:**
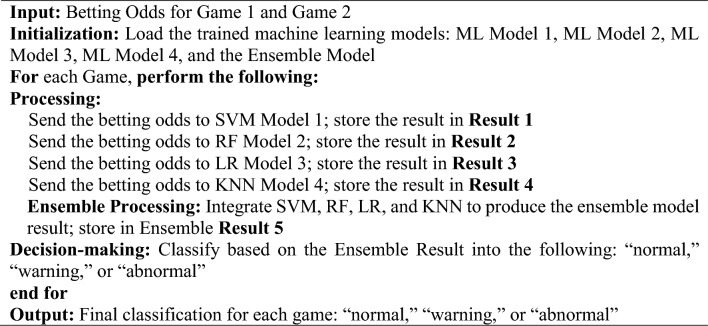
Algorithm 1 Ensemble process for detecting abnormal games

**Figure 2:**
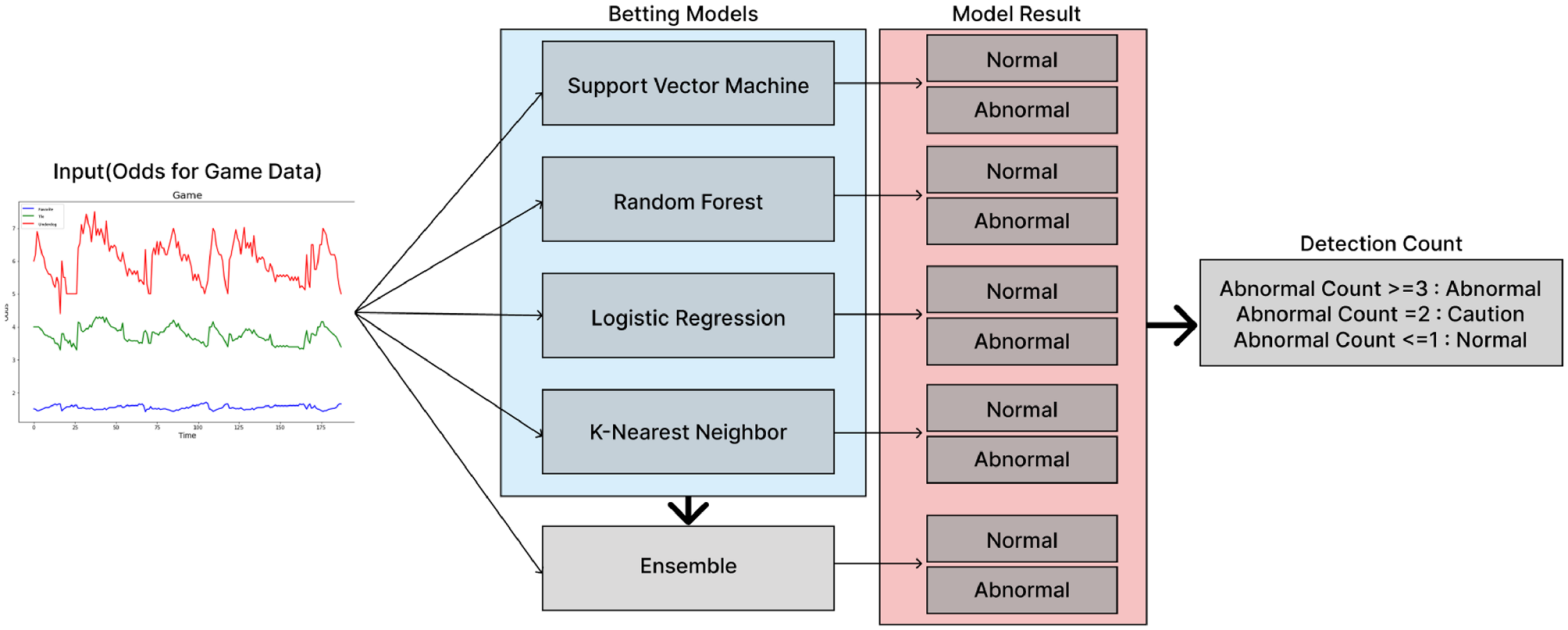
Betting anomaly detection process.

Developed a sophisticated multimodal artificial intelligence model designed to monitor and analyze different types of data for anomaly detection. The model has a process, shown in Fig. 2, that integrates input from multiple sources and uses an ensemble approach where each submodel is specialized for a specific data type. The system combines insights from these submodels to assess the overall situation and categorizes the results into different categories. The decision-making process is based on a consensus mechanism^28^. If the majority of sub-models flag an event as suspicious, the event is labeled as ‘abnormal.’ Consequently, the integrated model is capable of distinguishing between ‘normal’ and ‘abnormal’ outcomes with high accuracy. To provide more nuanced insights, the model categorizes the anomalies into three levels.

To illustrate the overall process of the model, want to detect anomalies in the odds data of a single match. Therefore, the odds data is classified into five models: four trained models and an ensemble model of four models. At this time, one odds data is input to five models as an input value, and each of the five models that received the data is judged as normal or abnormal, and five results are derived. At this time, if the count of Abnormal is 3 or more, it is Abnormal, 2 is Caution, and 1 or less is Normal. Therefore, each of the five classification models derives two prediction labels, but the overall model counts two prediction labels and derives a total of three results.

1. Normal: If the ‘Abnormal Count,’ which represents the number of sub-models indicating an anomaly, is less than 1, the situation is judged as normal, indicating typical and safe operational conditions.
2. Caution: If the ‘Abnormal Count’ is exactly 2, it indicates a need for caution. This level suggests that there might be potential issues or emerging risks that require closer monitoring or preventive measures.
3. Abnormal: If the ‘Abnormal Count’ is 3 or more, the situation is judged as abnormal. This classification signifies a high likelihood of a significant issue or anomaly that needs immediate attention and possibly corrective action.

This comprehensive dataset allowed us to identify patterns associated with abnormal matches, thereby enabling the classification model to learn and distinguish between normal and abnormal labels. Hence, the classification model was employed as a means to effectively analyze and comprehend the intricacies within the dataset. The data used for classification were employed to identify patterns of win odds, tie odds, and loss odds observed in soccer matches, using the proposed method. These patterns were then converted into specific values and utilized in the classification process.

#### Support vector machine

An SVM is a data classification model that uses a decision boundary to separate the data space into two disjoint half properties. New input data are classified based on their similarity to one of these properties. The larger the boundary data gap, the more accurate the classification model. It is, therefore, common to set up random outliers on both sides of the decision boundary, known as margins. In this study, a maximum margin was created to enhance classification accuracy, and the data entering the margin were eliminated^29^.

The SVM algorithm on the p-dimensional hyperplane is shown in Eq. (5), with $f\left( X \right) = 0$.

5 $$f\left( X \right) = \beta_{0} + \beta_{1} X_{1} + \cdots + \beta_{p} X_{p}$$

6 $$f\left( X \right) = 0$$

The $f\left( X \right)$ value on the hyperplane is 1 (Class1) if $f\left( {X_{i} } \right) > 0$, otherwise − 1 (Class2) if ($f\left( {X_{i} } \right) < 0)$. Data were considered well sorted when the value of Eq. (7) was positive, following $Y_{i}$ on (− 1, 1).

7 $$Y_{i} \left( {\beta_{0} + \beta_{1} X_{i1} + \cdots + \beta_{p} X_{ip} } \right) > 0$$

With a hyperplane, as shown in Eq. (7), the data can be divided by different angles. However, for a classification model to be highly accurate, the hyperplane should be optimized by maximizing the margin between different data points. This leads to finding the maximum “M” (margin), as shown in Eq. (9). Consequently, the hyperplane and margin are designated while allowing errors $\in_{i}$ to some degree, before eliminating all data inside the margin as outliers.

8 $$\beta_{0} ,\beta_{1} , \ldots ,\beta_{p} ,\; \in_{1} , \ldots , \in_{n} MMaximizeM$$

9 $$subject to \mathop \sum \limits_{j = 1}^{p} \;\;\beta_{j}^{2} = 1$$

10 $$Y_{i} \left( {\beta_{0} + \beta_{1} X_{i1} + \cdots + \beta_{p} X_{ip} } \right) \ge M\left( {1 - \in_{1} } \right)$$

11 $$\in_{1} \ge 0, \mathop \sum \limits_{i = 1}^{n} \;\; \in_{i} \le C$$

For SVM model, the C(Regularization Strength) value is 0.1 to prevent overfitting, and since the values of the data are linear, the kernel is linear, and abnormal matches of the odds do not have regular features, so RBF(Radial Basis Function) is adopted to derive such non-linear features^30^.

#### Random forest

In the RF model, decision trees—the hierarchical structure composed of nodes and edges that connect nodes—help determine the optimal result. A decision tree rotationally splits learning data into subsets. This rotation-based division repeats on the divided subsets until there is no more predictive value left, or the subset node’s value becomes identical to the target variable. This procedure is known as the top-down induction of decision trees (TDIDT), in which the dependent variable *Y* serves as the target variable in the classification; furthermore, vector v is expressed by Eq. (12).

12 $$\left( {v, Y} \right) = \left( {x_{1} ,x_{2} , \ldots ,x_{d} , Y} \right)$$

While classifying data using TDIDT, Gini impurity may be used to measure misclassified data in a set. While randomly estimating the class, a set with a likelihood of misjudgment near 0 is said to be pure. Therefore, Gini impurity enhances the accuracy of the RF model^31^.

13 $$I_{G} \left( f \right) = \mathop \sum \limits_{i = 1}^{m} f_{i} \left( {1 - f_{i} } \right) = \mathop \sum \limits_{i = 1}^{m} \left( {f_{i} - f_{i}^{2} } \right) = \mathop \sum \limits_{i = 1}^{m} f_{i} - \mathop \sum \limits_{i = 1}^{m} f_{i}^{2} = 1 - \mathop \sum \limits_{i = 1}^{m} f_{i}^{2}$$

Trees are trained to optimize split function parameters related to internal nodes, as well as end-node parameters, to minimize defined objective functions when *v* (data), S_0_ (trained set), and real data labels are provided. The RF model optimizes and averages the decision tree results using the bagging method before classification. Bagging or bootstrap aggregation—meaning simultaneously bootstrapping multiple samples and aggregating results from machine learning—is a method that averages diverse models to identify the optimized version.

Since the number of trees determines the performance and accuracy of the LF model, we ran Gridsearch with increasing numbers of trees, and found that the best performance was achieved with 50 trees. We also set the ratio to 0.4 to determine the maximum number of features in the tree, and the maximum depth of the tree to 10 to prevent overfitting^32^.

#### Logistic regression

LR is a supervised learning model that predicts the probability of given data belonging to a certain range between 0 and 1. The target variable is binary: 0–0.5 and 0.5–1. LR is linear, and each feature value multiplied by a coefficient and added by the intercept gives log-odds against the predicted value, enabling data classification. Therefore, the probability (P) of the event occurring or not occurring was calculated, and the log of the odds was calculated for the classification through the final value^33^.

14 $$Odds = \frac{{P\left( {event occurring} \right)}}{{P\left( {event not occurring} \right)}}$$

To evaluate the suitability of the results to the model, we must calculate and average the loss of the sample. This is referred to as log loss, expressed in Eq. (15), which contains the following elements: *m* = total number of data points, *y*^*(i)*^ = class for data *i*, z^i^ = log-odd of data *i*, and h(z^(i)^) = log-odds sigmoid that identifies a coefficient minimizing log loss, which gives the optimized model.

15 $$- \frac{1}{m}\mathop \sum \limits_{i = 1}^{m} \left[ {y^{\left( i \right)} loglog \left( {h\left( {z^{\left( i \right)} } \right)} \right) + \left( {1 - y^{\left( i \right)} } \right)log\left( {1 - h\left( {z^{\left( i \right)} } \right)} \right)} \right]$$

Once log-odds or property coefficient values were calculated, they could be applied to the sigmoid function to calculate the outcome of the data, ranging between 0 and 1 and belonging to a given class. In this study, a loss function was used to identify values near 0 or 1, to sort normal and abnormal matches.

In this study, to find the optimal hyperparameters for each of the four models, we used Gridsearch to fine-tune the weights of each model and select the model with the optimal accuracy. For the LR model, the C(Regularization Strength) value was set to 0.1 to prevent overfitting, and to normalize the data values, Lasso regression analysis was adopted, which can well judge the flow of a specific match, and regularization was performed using liblinear, which is suitable for small datasets for optimization^34^.

#### K-nearest neighbor

KNN is a classification algorithm of KNNs, based on their data label, using the Euclidean distance formula to evaluate the distance. Based on the Euclidean distance, d (distance) between A (x1, y1) and B (x2, y2) in a two-dimensional land is shown in Eq. (16).

16 $$d\left( {A,B} \right) = \sqrt {(x_{2} - x_{1} )^{2} + (y_{2} - y_{1} )^{2} }$$

To distinguish between normal and abnormal matches, the current study designated k as 2 and split array figures into normal or abnormal matches using the betting odds pattern appropriate for each match^35^. In this study, we set k = 2, as it involves the classification of two classes: normal and abnormal. Consequently, the issue of ties can arise when an equal number of nearest neighbors belong to different classes. To address this challenge, the analysis was performed by augmenting model stability through the utilization of k-fold cross-validation. This technique enables the evaluation of both the accuracy and stability of the classification model, ensuring a more robust and reliable classification outcome for cases in which k = 2. After determining the betting odds of a new match, the match array pattern allowed us to determine whether it was more normal or abnormal. For the KNN model, Gridsearch was conducted by adjusting the initial k value, and as a result, k = 2 was finally adopted. In addition, Manhattan distance, Minkowski distance, and Euclidean distance were used as distance metrics, but general Euclidean distance was adopted due to the complex nature of the data and the small number of data^36^.

### Data preprocessing

**Figure 3:**
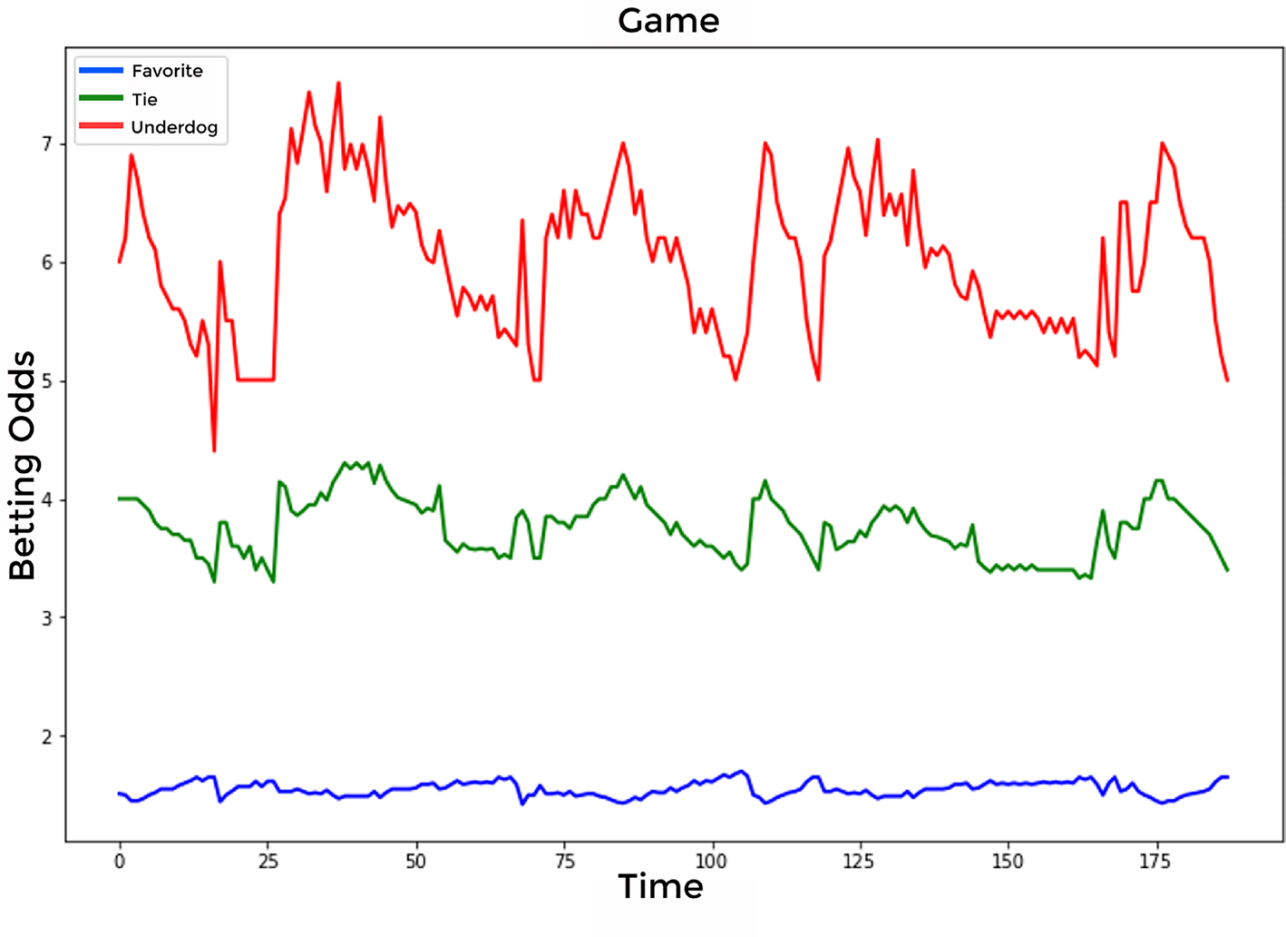
Betting odds graph of matches (One of 2607 match data).

This study used hourly win-tie-loss betting odds data to classify abnormal and normal matches. K-league football matches and match-fixing cases between 2000 and 2020 were used as data sources. The training data, spanning 20 years, is derived from K-League soccer matches, where each of the 12 teams plays 33 games. However, the dataset initially had a higher count of matches. Among these, a subset of matches was identified as having static data, characterized by minimal movement in betting odds due to low betting volumes. These matches were excluded from our analysis because their static nature does not provide useful insights for identifying betting trends. Consequently, the refined dataset for training consists of 2,607 data points. These points represent matches that attracted a significant number of bets, making them more relevant for our analysis in understanding betting patterns and trends. The learning data were based on 2586 normal and 21 abnormal matches. The matched dividend data are shown in Fig. 3. On the x-axis, representing “Time,” a value was assigned to each time flow. The win-tie-loss betting odds value was represented on the y-axis. Figure 3 is an example of the time series flow of odds for one out of 2607 games. In Fig. 3, the x-axis represents the “Favorite” betting odds, suggesting the probability of a team playing in their local stadium or one close to their home base. Conversely, “Underdog” denotes the betting odds of a team playing in an unfamiliar environment, potentially impacted by various factors such as different field dimensions, playing surfaces, and atmospheric conditions. The “Tie” on the y-axis signifies the betting odds of both teams tying due to an identical score in the match. Additionally, the x-axis represents the “Time” value in minutes. Thus, the evolution of betting odds is depicted as a time series, capturing the odds both before the soccer game began and as it progressed.

For data selection, using matches not identified as abnormal could result in an inaccurate model. Therefore, only matches confirmed as actual instances of abnormality in the K-League were examined and utilized for training as abnormal cases.

**Figure 4:**
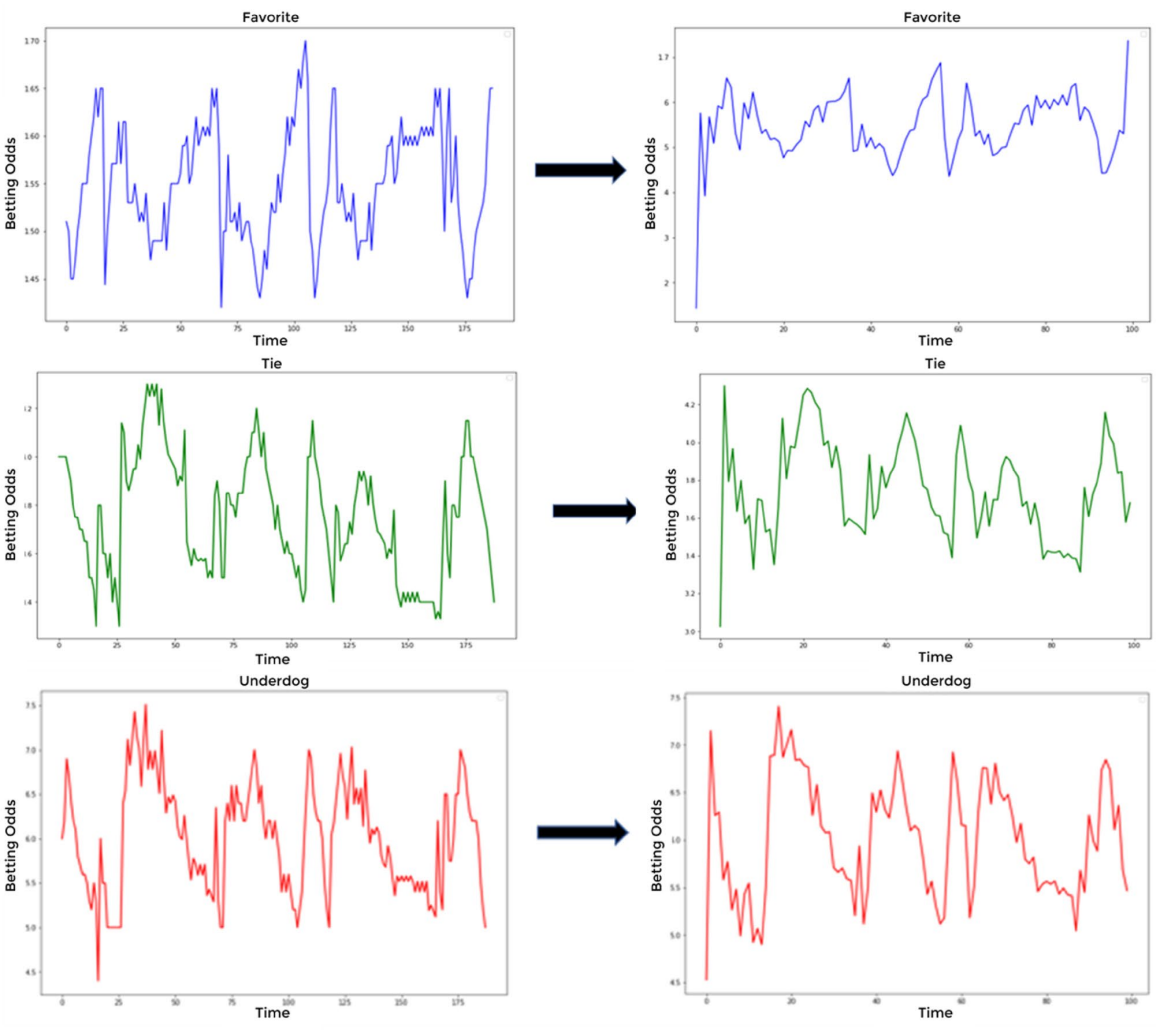
Data dimension synchronization.

Before learning, we checked whether the betting odds data and length of each match were irregular. For instance, there may be 50 data points for match A and 80 points for match B. In such a case, the difference in data dimensions hinders the model’s learning process. Therefore, data dimensions should be evened before learning. Given the average data length of 80 to 100, the length of every dividend datum was adjusted to 100 in an analyzable form, before smoothing and implementation by adding a Sin value. Figure 4 shows the data dimension adjustment to 100 without changing the overall betting odds graph pattern and the application of the Sin-based smoothing. Superimposing a Sin wave onto our adjusted data enabled us to highlight potential periodicities and enhance the model’s ability to capture these recurrent patterns. The Sin-based smoothing technique, when post-data dimension adjustments are applied, emerges as an instrumental approach, not only ensuring the mitigation of unwarranted noise and fluctuations but also amplifying latent periodic trends, thereby promoting data uniformity across matches. This, in turn, cultivates an environment conducive for models to discern principal trends over outliers and enhances their capability to generalize across diverse and unseen datasets, fortifying their overall predictive proficiency^37^.

**Figure 5:**
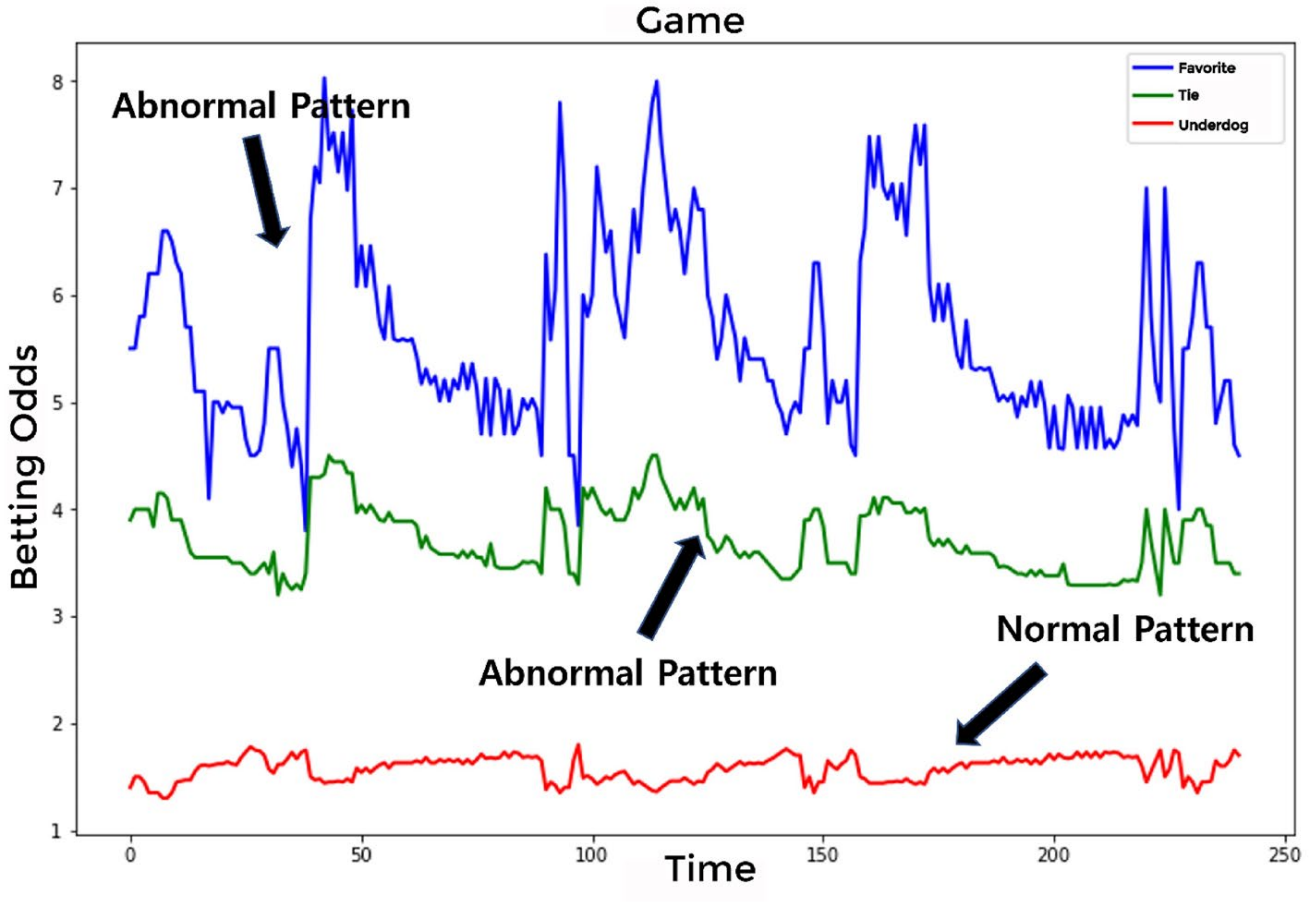
Abnormal match with both normal and abnormal patterns.

With the adjusted dimension of win-tie-loss betting odds data, Fig. 5 represents an abnormal match during learning, with no change in a given dividend. As shown in Fig. 4, each dimension was adjusted to the win-tie-loss betting odds data. When learning each of the win-tie-loss betting odds, Fig. 5 represents an abnormal match with no change in a given dividend, even for an abnormal match. However, its loss pattern can be considered a normal match. Consequently, the learning model can be considered a normal match when three different patterns are applied simultaneously.

**Figure 6:**
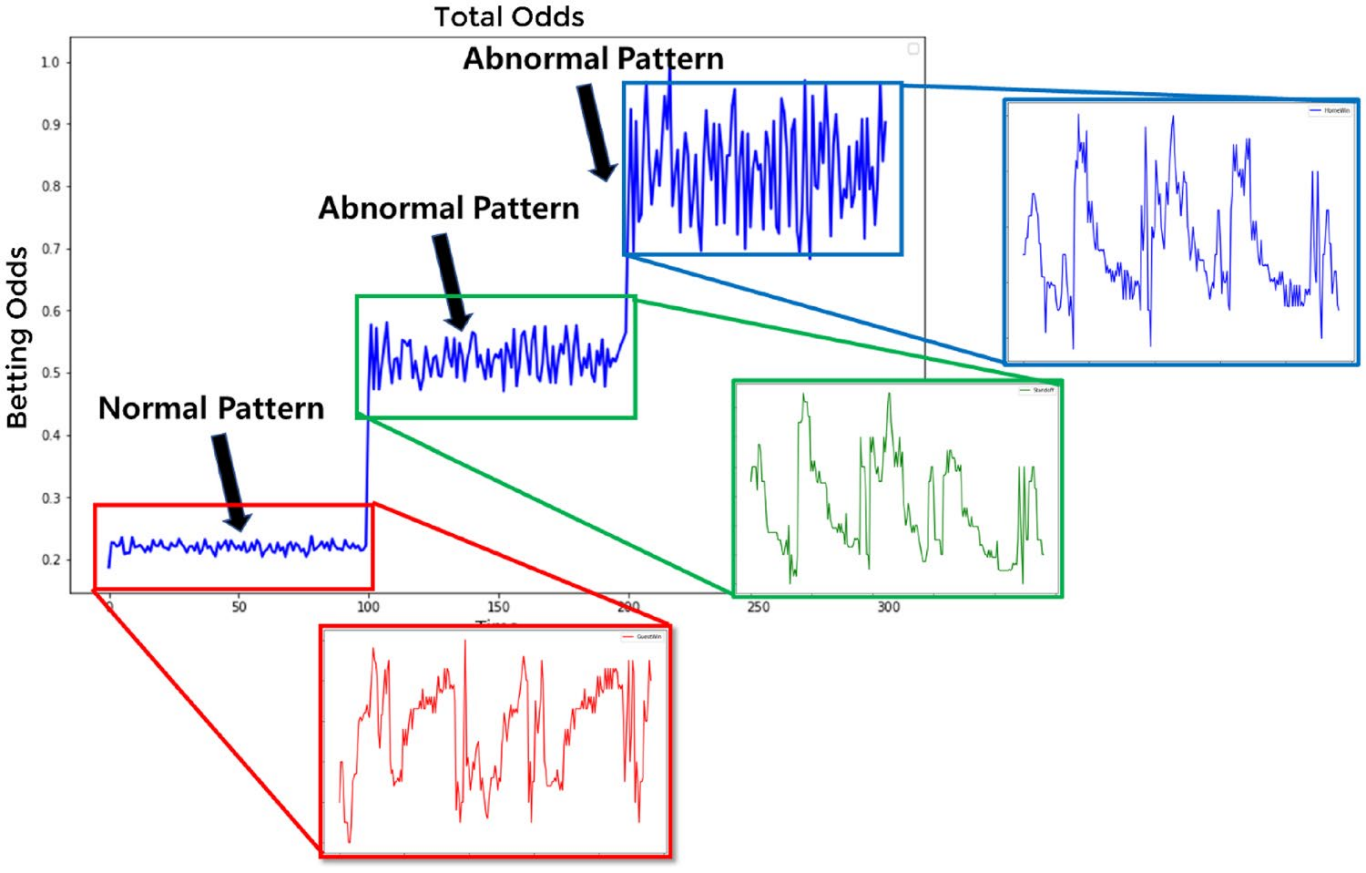
Total odds match dividend pattern.

To address this problem, datasets on win-tie-loss with a length of 100 each were converted to frame a single dataset of 300 in length. Figure 6 shows the result. Three types of betting odds, shown in Fig. 5, were combined to form a pattern, which in turn emphasized the characteristics of data-deprived abnormal matches during learning.

### Abnormal betting detection model

**Figure 7:**
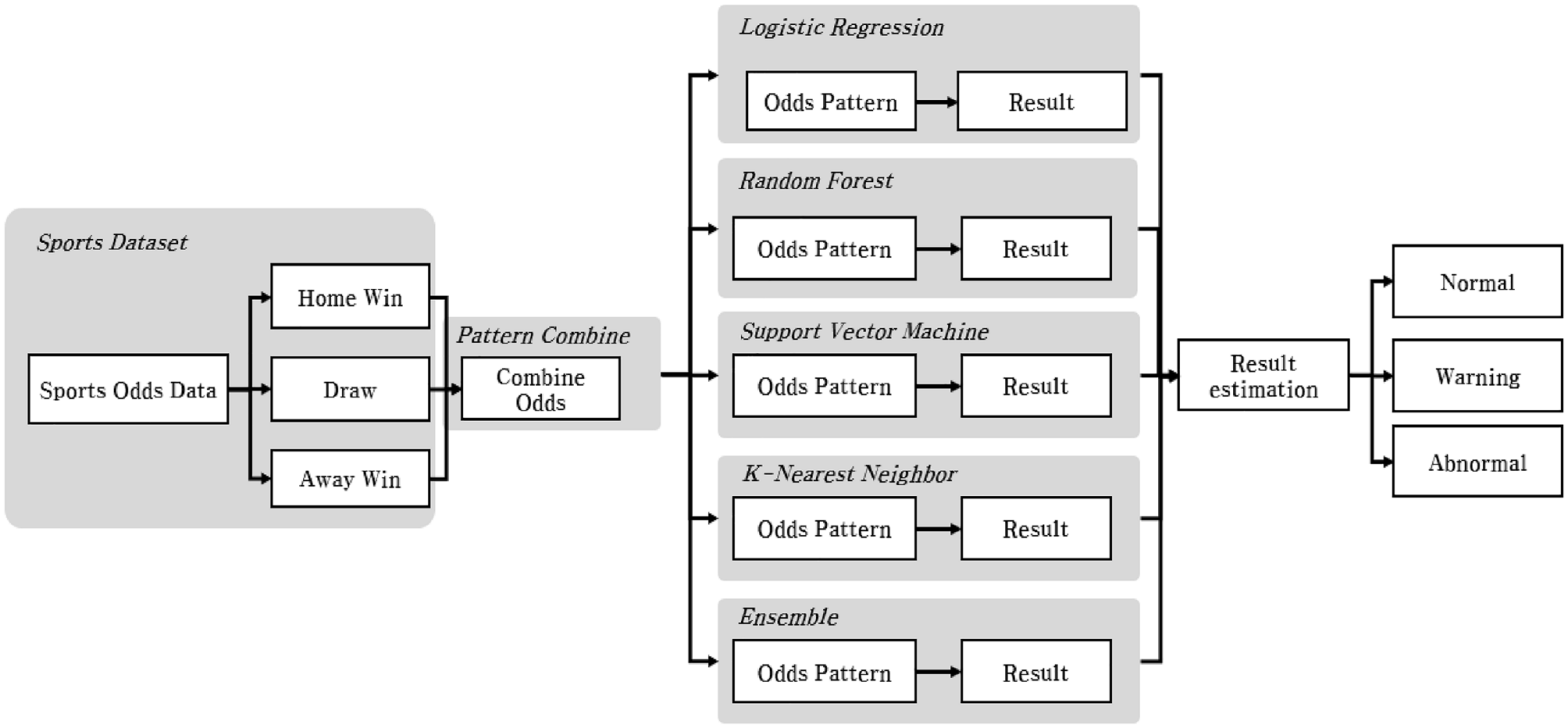
Process of the abnormal match detection model.

An abnormal match detection model was developed based on the data analysis results of each model, using the match result dividend data. Based on Fig. 7, the process begins with the real-time entry of data for each odds rate during a sports game. This input comprises three odds categories: Favorite, Tie, and Underdog. Subsequently, the input data undergoes a transformation through the Pattern Combine method proposed in this study, resulting in the generation of a new pattern. This newly derived pattern is then fed into the five learning models. Each model individually classifies games as normal or abnormal and provides corresponding results. Consequently, the outcomes of the betting patterns are obtained for each model, enabling a comprehensive evaluation of their performance. Based on the betting pattern analysis results from the five models, the abnormal betting detection model classified matches according to the number of abnormal matches as follows: one or less, normal; two, caution; three, danger; and four or more, abnormal. Figure 7 shows the models’ classification process, which provides a dividend pattern to help detect abnormal matches. In summary, we learned four machine learning models and created an ensemble model using the parameters of these four models, ultimately creating a total of five fraud detection models. We classified matches as normal, caution, dangerous, or abnormal based on the number of abnormal matches detected by all five models, rather than judging irregular matches based on each model’s results.

### Data analysis

The present study proceeded with machine learning using five performant multiclass models: LR, RF, SVM, KNN, and the ensemble model, which was an optimized version of the previous four models. This was used to classify normal and abnormal matches by learning their pattern from sports betting odds data. This study utilized the win, tie, and loss odds estimated by the iSports API using the 31 variables presented in Table 1.

Classification using the four models and one ensemble model used in the analysis shows high performance in judging data such as odds that do not have many variables. The accuracy of the training data for each model was 95% on average, and the loss value was 0.05 on average, which is a high accuracy for the training data. Therefore, the model was adopted to detect match-fixing.

As these 31 variables have an impact on the outcome of a soccer game, they were not directly employed as data; rather, their influence was reflected in the derived odds. Therefore, the odds variables for wins, ties, and losses were employed in this study. The dataset was sorted chronologically for wins, ties, and losses, irrespective of CompanyID (a variable used to differentiate and categorize the betting companies), and in cases of identical timestamps, averages were applied. Table 2 provides an explanation of the data subset. We collected betting data from three days before the start of the game until the end of the game. Data collection occurred whenever there was a change in Favorite, Tie, or Underdog betting data, without specifying a fixed time interval. Table 2 provides a detailed description of the variables used in this context. ScheduleID is a variable used to differentiate and identify specific matches. It allows us to distinguish details such as the match date and the teams involved in the game. In this research, CompanyID was utilized as a variable to distinguish among 12 different betting companies. Favorite, Tie, and Underdog represent betting data for wins, ties, and losses, based on the home team. These variables constitute the primary data used in this study, reflecting real-time changes in betting data. ModifyTime is a variable that records the time when data changes occurred. For example, if there were changes in betting data from Company A, the modified Favorite, Tie, and Underdog data would be recorded along with the time of the modification. If Company A experienced changes in betting data while Company B did not, only the modified betting data from Company A would be recorded.

**Table 2:** Data subset.

ScheduleID	CompanyID	Favorite	Tie	Underdog	ModifyTime
371333	23	2.21	3.2	3.15	2010-02-27 12:52:00
371333	35	2.17	3.2	3.25	2010-02-27 12:51:00
371333	35	2.19	3.2	3.2	2010-02-27 12:41:00
371333	35	2.26	3.2	3.1	2010-02-27 12:29:00
371333	35	2.28	3.2	3	2010-02-27 12:15:00
371333	23	2.25	3.2	3.1	2010-02-27 12:14:00
371333	35	2.25	3.2	3.1	2010-02-27 12:06:00
371333	35	2.21	3.2	3.2	2010-02-27 07:34:00
371333	23	2.23	3.2	3.15	2010-02-27 07:18:00
371333	35	2.2	3.2	3.2	2010-02-27 06:55:00
371333	35	2.17	3.2	3.3	2010-02-27 06:18:00
371333	23	2.16	3.2	3.3	2010-02-27 05:50:00
371333	23	2.15	3.2	3.3	2010-02-27 05:49:00
371333	23	2.16	3.2	3.3	2010-02-27 05:47:00
371333	23	2.15	3.2	3.3	2010-02-27 05:45:00
371333	35	2.2	3.2	3	2010-02-27 05:39:00
371333	23	2.17	3.2	3.3	2010-02-27 04:53:00
371333	35	2.14	3.2	3.15	2010-02-27 04:09:00
371333	35	2.1	3.2	3.25	2010-02-27 02:56:00
371333	23	2.13	3.2	3.4	2010-02-27 02:38:00
371333	35	2.05	3.2	3.35	2010-02-27 02:17:00
371333	23	2.09	3.2	3.5	2010-02-26 23:55:00

This study process involves testing five models using a dataset that consists of 2607 items. This dataset is utilized for learning purposes, comprising 2586 normal matches and 21 abnormal matches. For the validation phase, a separate set of 20 matches is employed, which is evenly divided into 10 normal and 10 abnormal matches. This setup ensures that the models are both trained on a comprehensive dataset and then accurately validated using a balanced mix of normal and abnormal match data. Acknowledge that the dataset in our study may be perceived as limited in quantity; however, as we deal with betting data on unusual matches, in practice, we cannot use data without verified instances of matches with illegal odds for training. This is because if the model is trained with abnormal match odds that are actually from a normal match, there is a problem. Therefore, only data verified with actual cases were used. Although the size of the learning dataset is small, it contains all the patterns of illegal/abnormal games that occur within it; therefore, it represents the phenomenon or pattern studied in this research. The RF, KNN, and ensemble models recorded a high accuracy of over 92%, while the LR and SVM models were approximately 80% accurate (Table 3).

**Table 3:** Model results.

Model	Thresh	Accuracy	Sensitivity	Specificity
LR	0.5	0.817	0.857	0.817
	0.01	0.788	0.714	0.789
SVM	0.5	0.784	0.857	0.784
	0.008	0.778	0.571	0.780
RF	0.5	0.932	0.429	0.937
	0.01	0.799	0.857	0.799
KNN	0.5	0.92	0.118	0.980
	0.01	0.86	0.318	0.901
Ensemble	0.5	0.931	0.054	0.990
	0.008	0.934	0.155	0.993

## Results

Five models were tested using data from 20 matches (10 normal and 10 abnormal). K-league football matches and match-fixing cases between 2000 and 2020 were used as data sources. In this study, the term “abnormal match” refers to games of match-fixing that occurred between the years 2000 and 2020 and resulted in actual legal punishment. “Normal match” refers to the remaining matches in the K-League dataset collected. Additionally, for model validation, the matches used were extracted through random sampling. Based on the betting odds of 20 matches, the classification performance of the model was evaluated using a confusion matrix, as presented in Table 3. Normal, caution, and abnormal results were classified using the four models of LR, SVM, RF, and KNN, while ensemble values of the models were determined by analyzing the total as the fifth result. In this approach, a game was classified as abnormal if the class assigned by each model contained four or more abnormal cases. Likewise, if three cases were classified as cautions, the game was classified accordingly.

The confusion matrix in Table 4 presents the actual values of normal and abnormal from actual data, and the predicted values were defined as normal, cautions, and abnormal. Of 10 normal matches, 8 were deemed valid, while the remaining 2 matches were rated as cautions in the LR, RF, and ensemble models. Out of 10 abnormal matches, 6 were valid, 2 were rated cautions, and 2 were rated as normal. Regular betting odds patterns in abnormal matches would have generated such decisions. Consequently, the model in the current study was 80% accurate for normal matches and 60% accurate for abnormal matches, owing to the lack of abnormality data, which prevented the model from accurately estimating irrelevant results. The proposed abnormal betting detection model proposed in this study is estimated to have an approximate 80% accuracy in identifying abnormal matches. In contrast to our model, the five pre-existing models failed to categorize the warning group, instead only distinguishing between normal and abnormal games. This resulted in a high likelihood of erroneously categorizing regular games as abnormal. Furthermore, when evaluating the performance of each model in classifying normal and abnormal games, the average accuracy ranged between 60 and 70%. Thus, the model introduced in this study not only provides a more nuanced classification via the caution category but also outperforms other models in terms of accuracy.

**Table 4:** Confusion matrix.

Actual values	Predictive values		
	Normal	Caution	Abnormal
Normal (Abnormal betting detection model)	8	2	0
Abnormal (Abnormal betting detection model)	6	2	2
Normal (SVM)	5	–	5
Abnormal (SVM)	9	–	1
Normal (RF)	7	–	3
Abnormal (RF)	6	–	4
Normal (LR)	8	–	2
Abnormal (LR)	7	–	3
Normal (RF)	6	–	4
Abnormal (RF)	7	–	3
Normal (Ensemble)	6	–	4
Abnormal (Ensemble)	5	–	5

Moreover, after collecting data from real-time matches, we applied five models to construct a system capable of detecting match-fixing in real time. The models are built on previous match data and collect real-time match data to ascertain fraudulent matches. Our study aimed to provide an environment for real-time analysis and investigation by building a system that collects real-time data before and during matches, decides whether a match is suspicious, and acts promptly.

However, previous research on data-based statistical detection of match-fixing revealed that match-fixing cases are relatively minor compared to normal matches^18^, which the current study confirmed. Real-time data collection on sports matches could contribute to the creation of a more accurate detection system.

Determining whether a match is fixed cannot rely solely on abnormal patterns and data^22^. However, the detection model could help identify abnormal and normal matches in real time and provide more detailed data to facilitate the investigation of match-fixing cases. Moreover, it could benefit the public, as these real-time data would detect match-fixing in games. Furthermore, the detection model may prevent match-fixing brokers and players from committing match-fixing, as they are aware of the risk of real-time detection. The results of this study could guide the future detection of match-fixing in sports.

## Discussion

In the realm of sports, match-fixing issues tend to occur constantly and damage the fundamental value of fairness in sports. Various methods have been proposed to solve this problem. Efforts have been made in sports to build a match-fixing anomaly-detection model using match data.

This study utilized a predefined criterion to distinguish between normal and abnormal matches in order to establish a system. Abnormal matches were defined as those that have been officially recognized as cases of match-fixing and have faced legal consequences. This definition ensures that the research results can identify actual instances of match-fixing. To validate the results obtained through data analysis, K-League soccer match data were utilized in this study. A total of 20 game data were used for validation, with 10 matches classified as normal and 10 matches classified as abnormal. The validation data were randomly sampled to ensure data diversity and representativeness, thereby enhancing the generalizability of the research results. For the validation of abnormal matches, a criterion was established based on the number of models among the five utilized models that categorized a match as abnormal. Depending on the number of models that classified a match as abnormal, matches were categorized as safe, caution, risk, or abnormal. This approach enabled the evaluation and validation of the reliability of the proposed models in identifying abnormal matches. Our ensemble method diverges significantly from traditional models, offering an enhanced prediction capability by synergistically integrating parameters from multiple individual models. This comprehensive approach allows for a richer capture of data nuances often overlooked by singular models. While traditional ensembles inherently enhance data generalization, our unique combination of four models amplifies resistance to overfitting, ensuring consistent performance across varied data terrains. This methodology, bolstered by inputs from five distinct models, not only acts as a shield against biases but also introduces an innovative “warning” category. This added layer aids in nuanced decision-making and provides stakeholders with a refined perspective to decipher borderline or ambiguous predictions.

In the academic world, studies have attempted to detect match-fixing using anomalous match data. Kim et al.^26^ converted sports dividend odds data into graphs and applied the CNN algorithm to sort normal and abnormal matches by comparing their dividend odds graphs. Ötting et al.^24^ used the GAMLSS model based on dividend odds and betting volume data to identify differences between fixed and non-fixed matches and evaluated the model’s ability to detect fixed matches.

Previous studies have examined suspected matches using a single model based on football match dividend odds data, with an accuracy rate of 70–80%. The misclassification rate was approximately 20%. However, inevitable biases and errors in single-model analyses hinder their practical application. Consequently, the current study aimed to suggest a solution to sports match-fixing using various AI models to detect anomalies based on dividend odds by constructing a database with such variables as sports match results, league ranking, and players.

To reduce errors in a single model, this study relied on four models frequently used in machine learning: LR, RF, SVM, and KNN classification. In addition, this study used the ensemble model, which is an optimized model of the previous four. Using these five models, this study aims to distinguish between normal and abnormal matches. The accuracy of the present results was higher than those in previous research for sorting matches, with three models (RF, KNN, and ensemble) showing an accuracy of over 90% and two (LR and SVM) models showing an accuracy of 80%. A combination of the models was used to identify suspicious matches, as each model suggests different suspicious cases, reducing the likelihood of considering valid matches as suspicious.

However, it must be acknowledged that the verification data in this study were limited in size, which precluded testing across diverse scenarios. This limitation may have stemmed from the constrained sample size. Nonetheless, a power analysis indicated that our sample size possessed an 80% power to detect the observed effect size. Furthermore, it must be noted that this study is fundamentally exploratory and stands among the pioneering efforts in this domain.

Another crucial aspect that must be emphasized is that our study utilized only actual, real-world data. When a match is flagged as irregular, it serves as concrete evidence of misconduct. Such fraudulent activities are highly sensitive and present substantial challenges in large-scale data collection. Furthermore, even if an unusual pattern emerges in a typical match, its value as verification data diminishes unless it can be confirmed as an irregularity resulting from foul play. We are fully aware of these constraints.

In the future, more real-world instances deemed as irregular matches should be collected to enhance precision in identifying abnormal games through iterative model refinements. Nonetheless, despite the disclosed limitations of our study, we are confident that our findings provide valuable insights in this field, laying the groundwork for more expansive subsequent studies.

## Conclusion

This study aimed to develop an AI-based sports match-fixing detection system using sports betting odds. The conclusions of this study are as follows.

First, five models were utilized to implement the system in this study. Specifically, four classification models—LR, RF, SVM, and KNN—were trained, and an ensemble model combined their optimal results. Three models (RF, KNN, and ensemble) achieved an accuracy of over 90%, while two models (LR and SVM) demonstrated an accuracy of approximately 80%.

Second, real-time match data were collected and the five models were applied to build a system to detect match-fixing in real time. The performance of the developed system was validated using 10 normal matches and 10 abnormal matches. The results showed an accuracy of 80% for normal matches and 60% for abnormal matches.

This study aimed to provide an effective preventive measure—an AI-based system—against match-fixing, in a context in which match-fixing undermines sports fairness and has a negative impact on the sports industry. The anomaly detection model utilizing real-time data can evaluate matches in real time and detect match-fixing, thereby benefiting the general public. Additionally, by raising awareness among match-fixing brokers and players of the risks associated with real-time detection, match-fixing can be prevented. The development of a system capable of detecting covert match-fixing in advance holds significant importance. Future research efforts are expected to expand this system to various leagues through the inclusion of data from abnormal matches or match-fixing incidents.

## Data Availability

The currently researched and/or analyzed betting dataset is not publicly available, as the raw data is currently confidential. However, the datasets available from the corresponding author (Ji-Yong Lee, 302479@knsu.ac.kr) on reasonable request. Furthermore, Information on K-League betting and match results can be found on the website of a betting company operated by the Korean government (https://www.betman.co.kr).
